# Genetic Variation Related to High Elevation Adaptation Revealed by Common Garden Experiments in *Pinus yunnanensis*

**DOI:** 10.3389/fgene.2019.01405

**Published:** 2020-02-11

**Authors:** Yan-Qiang Sun, Wei Zhao, Chao-Qun Xu, Yulan Xu, Yousry A. El-Kassaby, Amanda R. De La Torre, Jian-Feng Mao

**Affiliations:** ^1^ Beijing Advanced Innovation Center for Tree Breeding by Molecular Design, National Engineering Laboratory for Tree Breeding, Key Laboratory of Genetics and Breeding in Forest Trees and Ornamental Plants, Ministry of Education, College of Biological Sciences and Technology, Beijing Forestry University, Beijing, China; ^2^ Key Laboratory for Forest Resources Conservation and Utilization in the Southwest Mountains of China, Southwest Forestry University, Kunming, China; ^3^ Department of Forest and Conservation Sciences, Faculty of Forestry, The University of British Columbia, Vancouver, BC, Canada; ^4^ School of Forestry, Northern Arizona University, Flagstaff, AZ, United States

**Keywords:** elevation adaptation, RNA-seq, *F*_ST_ outlier, flavonoid biosynthesis, nucleotide diversity

## Abstract

Local adaptation, adaptation to specialized niches and environmental clines have been extensively reported for forest trees. Investigation of the adaptive genetic variation is crucial for forest resource management and breeding, especially in the context of global climate change. Here, we utilized a *Pinus yunnanensis* common garden experiments established at high and low elevation sites to assess the differences in growth and survival among populations and between the two common garden sites. The studied traits showed significant variation between the two test sites and among populations, suggesting adaptive divergence. To detect genetic variation related to environment, we captured 103,608 high quality SNPs based on RNA sequencing, and used them to assess the genetic diversity and population structure. We identified 321 outlier SNPs from 131 genes showing significant divergence in allelic frequency between survival populations of two sites. Functional categories associated with adaptation to high elevation were found to be related to flavonoid biosynthesis, response to UV, DNA repair, response to reactive oxygen species, and membrane lipid metabolic process. Further investigation of the outlier genes showed overrepresentation of the flavonoid biosynthesis pathway, suggesting that this pathway may play a key role in *P. yunnanensis* adaptation to high elevation environments. The outlier genes identified, and their variants, provide a basic reference for advanced investigations.

## Introduction

In harsh environment such as in high elevations, natural selection may result in changes in allele frequency to maximize fitness (Hoffmann and Sgrò, 2011). Understanding the influence of natural selection on genomic variation in natural populations, and identifying the adaptive loci have received increased attention in the field of adaptive evolution and evolutionary ecology (Tiffin and Ross-Ibarra, 2014; Flood and Hancock, 2017). Organisms are adapted to diverse habitats. Highlands are characterized by intense ultraviolet radiation, low temperatures, hypoxia, and reduced pathogen incidence, providing a unique environment to study adaptation to high elevation. Survival at high elevation environments is challenging and native plants and animals have developed effective strategies through specific morphological and physiological adaptations (Monge and Leon-Velarde, 1991; Alonso-Amelot, 2008; Storz et al., 2010; Gong et al., 2018; Halbritter et al., 2018). For example, maize plants growing at high elevation often accumulate flavonoids in their leaves and silks as a mechanism for coping with high levels of UV-B exposure (Zhang et al., 2003; Casati and Walbot, 2010). The recent development of high-throughput sequencing technologies has greatly accelerated the identification of key genes and genomics research, significantly promoting the research of adaptive evolution and ecology on non-model organisms (Stapley et al., 2010; Ekblom and Galindo, 2011), including conifers (Yeaman et al., 2016; Lind et al., 2018; De La Torre and Neale, 2019; De La Torre et al., 2019; Lu et al., 2019; Tyrmi et al., 2019).

*Pinus*, with >100 species, is the largest conifer genus with widespread natural distribution ranging from arctic and subarctic to subtropical and tropical regions in the Northern Hemisphere (Richardson, 1999; Farjon and Farjon, 2010). Pines display diverse mountainous adaptability, with parapatric closely related species continuously distributed across varying elevation gradients (Kuan, 1981; Richardson, 1999; Farjon and Farjon, 2010). Although pines span a large elevational range, the genetics of adaptation to high elevation is still poorly understood. *Pinus yunnanensis* is the dominant pine in southwest China with a continuous distribution in the Yunnan–Guizhou region at elevations ranging from 700 to 3,000 m above sea level (m.a.s.l.) (Wu, 1956; Mao and Wang, 2011). *P. yunnanensis* morphological variation is significant across its range, and regions divided by mountain chains featuring different climatic conditions (Yu et al., 1998; Yu et al., 2000; Mao et al., 2009). Recent analysis based on maternally inherited mitochondrial (mt) and paternally inherited chloroplast (cp) DNA markers found continuous genetic differentiation over the majority of its range, and discrete isolated local clusters in the northwest and east peripheries. The discrete differentiation between the two genetic groups is coincident with their niche divergence and geographical isolation (Wang et al., 2013).

The genus *Pinus* and other conifers are known for their exceedingly large and complex genomes, varying from 16 to 35 Gbp (De La Torre et al., 2014; Leitch et al., 2019). Despite the decreasing costs of sequencing, whole-genome resequencing of large numbers of pine individuals is still not feasible. RNA sequencing (RNA-seq), in which the expressed part of genome is sequenced, represents a powerful alternative to whole-genome sequencing, allowing the genotyping of thousands of loci for non-model species with large genomes (Schlötterer et al., 2014; Hoban et al., 2016; Jones and Good, 2016; Oney-Birol et al., 2018). The draft genome of *Pinus taeda* (Wegrzyn et al., 2014; Zimin et al., 2014) may be used as a reference genome, and assist a reference-based RNA-seq approach to genotype expressed gene regions for population genomic studies.

In this study, we aim to discover the genetic variation related to adaptation to high elevation environments in *P. yunnanensis,* by comparing the survival of populations from common garden experiments in high and low elevations. Hundreds of thousands of single nucleotide polymorphisms (SNPs) from expressed regions including 8,595 genes were captured using RNA-Seq. Genetic diversity and population structure were resolved. SNPs, genes and pathways related to adaptation to high elevation environments were identified based on *F*_ST_ outlier analyses.

## Materials and Methods

### Sampling and Transcriptome Sequencing

**Figure 1:**
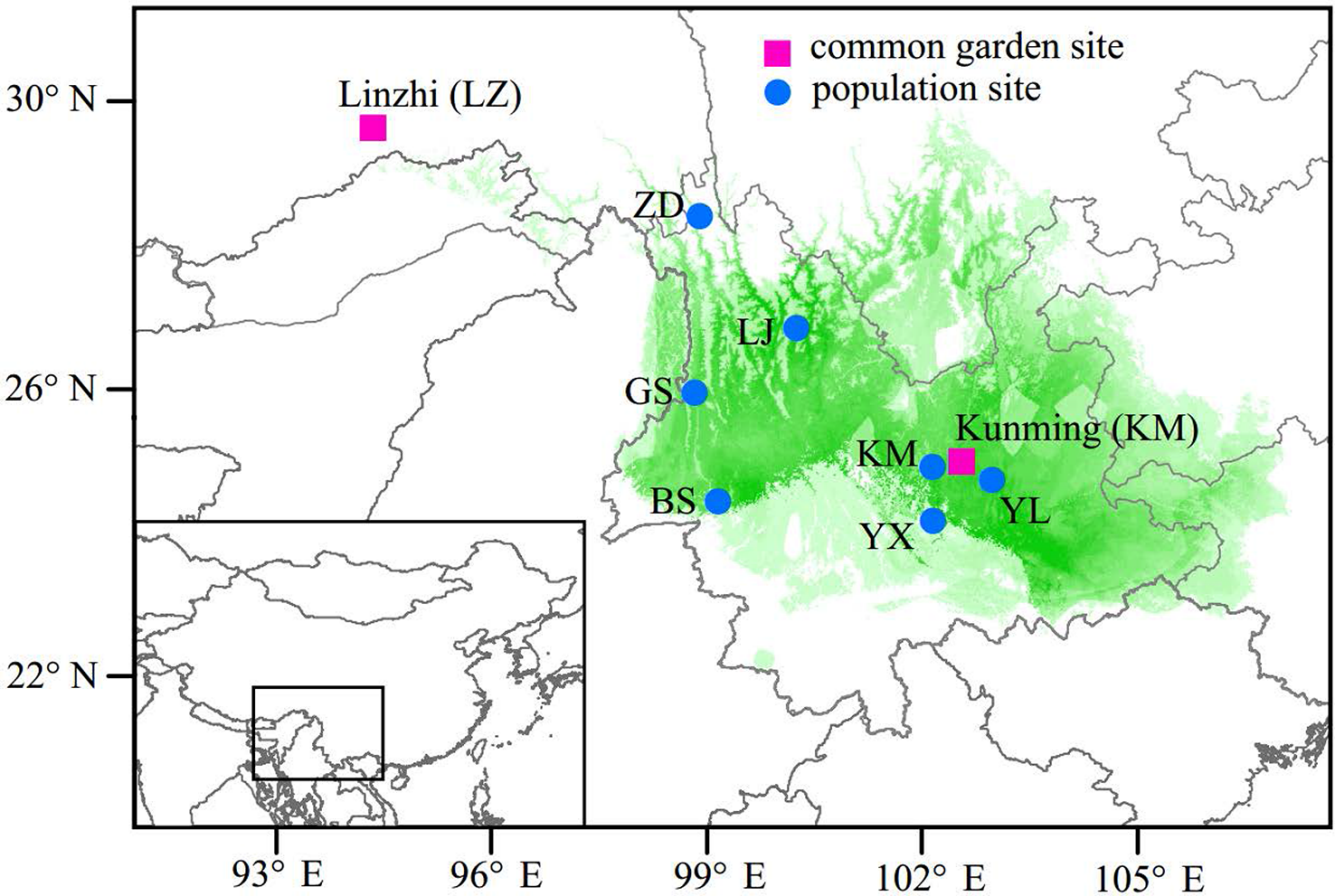
Geographic origins of the sampled populations and locations of the two common garden experiments. Green coloring illustrates the potential distribution of *P. yunnanensis* as suggested by Mao and Wang (2011).

In the spring of 2011, two *P. yunnanensis* common gardens were established at a high altitude site in Linzhi (LZ, 2,950 m.a.s.l.), Tibet and a low elevation site in Kunming (KM, 1,890 m.a.s.l.) (**Figure 1**), Yunnan, China. The LZ site (29°40′N, 94°20′E) is the native habitat for *Pinus densata*, a close relative of *P. yunnanensis*, and represents a high elevation environment characterized by cold and strong UV, with mean coldest-month temperature of –3.1°C, mean warmest temperature of 14.5°C, mean annual temperature of 6.5°C, average annual precipitation of 785 mm, and 185 frost-free days. While, the KM site (25°04′N, 102°46′E) is located in the central distribution of *P. yunnanensis* and characterized by mild, moist, and low seasonality climate, with mean coldest-month temperature of 8.1°C, mean warmest temperature of 20.2°C, mean annual temperature of 15°C, average annual precipitation of 1,035 mm and 334 frost-free days. Bulked seeds from 7 natural populations (LJ, YL, BS, GS, YX, ZD, KM) were collected to represent *P. yunnanensis’* wide range of natural genetic variation (**Table 1**). The two common gardens were established with random block designs, with 50 or 60 seeds of each population in each block and four or five blocks for each site (LZ and KM). Survival rate (measured at 2017) and seedling height (measured at 2016) of each population were measured in the 6-7^th^ year. In the high elevation site (LZ), all surviving trees (only five individuals) originated from the KM population were sampled (KM_(LZ)_). Additionally, we randomly selected 49 individuals representing 5 BS_(KM)_, 4 GS_(KM)_, 10 LJ_(KM)_, 10 YL_(KM)_, 5 YX_(KM)_, 5 ZD_(KM)_, and 10 KM_(KM)_ as representatives of the low elevation site (KM) (**Tables 1** and **S1**).

**Table 1:** Geographic origin and sample size of the sampled *Pinus yunnanensis* populations for RNA-seq.

Population	Population code	Longitude (E)	Latitude (N)	Altitude (m)	Sample size
Lijiang (LJ)	LJ_(KM)_	100°13′	26°53′	2,493	10
Yiliang (YL)	YL_(KM)_	103°10′	24°43′	1,846	10
Baoshan (BS)	BS_(KM)_	99°08′	24°28′	1,897	5
Gongshan (GS)	GS_(KM)_	98°49′	25°58′	1,616	4
Yuxi (YX)	YX_(KM)_	102°09′	24°15′	1,849	5
Zhongdian (ZD)	ZD_(KM)_	99°32′	28°09′	3,048	5
Kunming (KM)	KM_(KM)_	102°37′	24°58′	2,242	10
	KM_(LZ)_				5

Fresh needles from current year branches of all 54 sampled individual trees were collected for RNA-seq in June 2017, and immediately placed in liquid nitrogen and stored at –80°C until further use. Total RNAs were extracted using RNAprep pure Plant Kit (Tiangen, Beijing, China) according to the manufacturer’s protocols. NanoDrop 2000 Spectrophotometers and Agilent 2100 Bioanalyzer were used to evaluate RNA concentration and integrity. NEBNext Ultra RNA Library Prep Kit (New England BioLabs) was used for cDNA library construction. Briefly, the mRNA purification was performed with magnetic oligo (dT) beads using a Dynabeads mRNA Purification Kit (Invitrogen). The mRNA fragmentation was implemented using RNA fragmentation Kit (Ambion, Austin, TX, USA). Random hexamer primers and reverse transcriptase (Invitrogen) were used to synthesize the first-strand cDNA. Subsequently, DNA polymerase I (New England BioLabs) and RNaseH (Invitrogen) were used to synthesize the second-strand cDNA. Adapter was ligated to the double strand cDNA fragments with a single ‘A’ addition after end repair. Approximately 450 bp cDNA fragments were selected using Ampure XP beads (Beckman). The selected cDNA fragments were PCR amplified to complete library preparation. The concentration and fragment size of the cDNA library were assayed using Real-Time PCR system and Agilent 2100 Bioanalyzer, respectively. The library was sequenced on an Illumina HiSeq X Ten sequencing platform to generate 150 bp paired end raw reads.

### Sequence Quality Control and Mapping

Raw reads were filtered and trimmed using Trimmomatic (Bolger et al., 2014) to remove adapter sequences and low-quality bases (Phred quality <20) from either the start or the end of the reads. After trimming, reads shorter than 36 bases were completely discarded. FastQC (http://www.bioinformatics.babraham.ac.uk/projects/fastqc/) was used to assess the quality of the raw and clean sequence data. Clean reads from each sample were mapped to the *P. taeda* reference genome v1.01 (Wegrzyn et al., 2014; Zimin et al., 2014) using STAR (Dobin et al., 2013). Single nucleotide polymorphism (SNP) calling was first performed using SAMtools (Li et al., 2009) and BCFtools (Li et al., 2009) with default settings. Based on the distribution of SNPs gained in the first calling, a reduced reference genome consisting of all 57,783 scaffolds with at least 1 SNP with missing rate <90% was created to decrease the computational load in the following steps. Reduced sequence alignment (in BAM format) files where only conveying reads mapped to the reduced reference genome were generated for SNP calling using the Genome Analysis Tool Kit (GATK v3.7-0) (DePristo et al., 2011). The command, “AddOrReplaceReadGroups” from GATK was used for adding of read group information and further sorting. PCR duplicates were removed using “MarkDuplicates” from Picard (http://broadinstitute.github.io/picard/). A GATK command, “SplitNCigarReads”, was used to split reads spanning splice junctions, and reassign mapping qualities to all good alignments. To minimize the mis-alignment of bases around insertions and/or deletions (indels), local realignment around indels was performed using RealignerTargetCreator and IndelRealigner from GATK. The final BAM files were produced from the local realignment for further analysis.

### Variant Calling and Filtering

SNP calling was performed with “HaplotypeCaller” from GATK to produce a genomic variant call format (gVCF) file for each sample, and “GenotypeGVCFs” from GATK was then used to perform the multi-sample genotyping, which produced a raw set of joint SNPs and indels. Several filtering steps were used to minimize the number of false positive SNPs and to retain high-quality SNPs: (1) we kept only biallelic SNPs with at least 5 bp away from any indels; (2) GATK hard filtering was applied to remove SNPs with a criteria of RMSMappingQuality (MQ) < 40.0, QualByDepth (QD) < 2.0, FisherStrand (FS) > 30.0, StrandOddsRatio (SOR) > 4.0, MappingQualityRankSumTest (MQRankSum) < –12.5 and ReadPosRankSumTest (ReadPosRankSum) < –8.0; (3) SNPs with genotype quality (GQ) < 20 and depth (DP) < 10 in a single individual were treated as missing data, and we removed those SNPs with missing rate > 20%; and (4) SNPs with minor allele frequency (MAF) < 5% were removed. The remaining 103,608 SNPs were used in downstream analysis.

### Functional Annotation of Outlier SNPs

We built a local database based on released gene annotation for *P. taeda* genome v1.01 using SnpEff (version 4.3T) (Cingolani et al., 2012). Subsequently, functional annotation for each SNP was predicted with putative functional effects defined in the SnpEff. All SNPs were partitioned into 5'UTR, coding sequence (CDS), intron, 3'UTR, splice site, and intergenic mutations. Further we categorized SNPs within CDS as synonymous and nonsynonymous categories. Plant transcription factors (TFs) database, PlantTFDB 4.0 (http://planttfdb.cbi.pku.edu.cn/), were used to retrieve TFs annotations.

### Population Structure and Genetic Diversity

Principal component analysis (PCA) was performed using the R package SNPRelate (version 1.16.0) (Zheng et al., 2012). ADMIXTURE (version 1.3.0) (Alexander et al., 2009) was run to infer the population structure, with the number of genetic clusters (*K*) ranging from 2 to 5, and 10 replicates were run for each *K*. The most likely *K* value was identified by minimizing the cross-validation error evaluated in the 10-fold cross-validation procedure. To eliminate the effect of linkage disequilibrium (LD), we thinned the SNP set to select one SNP from each interval of 5 kb and run ADMIXTURE under both all SNP (103,608) and thinned SNP (18,329) sets.

We calculated average pairwise estimates of the number of nonsynonymous substitutions per nonsynonymous site (d_N_), synonymous substitutions per synonymous site (d_S_), and their ratio (d_N_/d_S_) for individual genes. Nucleotide diversity (π) was calculated for each gene using VCFtools (Danecek et al., 2011).

### Identification of Outlier SNPs

Based on the population structure obtained by PCA and ADMIXTURE results, the population BS was excluded in subsequent analyses given its different genetic composition. To identify outlier SNPs associated with high vs. low elevation adaptation, we conducted population comparison (KM_(LZ)_ *vs*. KM_(KM)_ and YL_(KM)_) between the high-elevation selected population (KM_(LZ)_) in high elevation site (LZ) and the survival populations from Kunming (KM) and Yiliang (YL) in the low elevation site in KM (KM_(KM)_ and YL_(KM)_). Here, populations from KM and YL were in an identical genetic cluster, hereafter named KM-YL_(KM)_. In addition, a second comparison, KM_(LZ)_ *vs*. ALL_(KM)_ which included 6 survival populations in Kunming site excluding population BS, was performed with the aim of providing more information for reference, regardless of the impact of population structure which may potentially cause false positives. Population differentiation (*F*_ST_) values of each SNP were calculated using VCFtools (Danecek et al., 2011).

Outlier SNPs were determined by combining *F*_ST_ scan with a randomization procedure that involving repeated drawing of random samples (100 times). First, SNPs with the top 1% *F*_ST_ values were selected as putative outlier SNPs for each comparison. Next, we removed the putative outlier SNPs that are likely to be false positives found by chance when defining population comparisons with randomization procedure. For each population comparison, 100 permutations were performed to produce 100 randomized population comparisons by randomly re-sampling the same number of individuals from the original sample set. Then, putative outlier SNPs were also detected respectively for each randomized population comparison. Finally, for each putative outlier SNP from the real population comparison, we retained only those with a recurrence rate less than 0.01 to generate the final set of outlier SNPs.

### Functional Enrichment Analysis

Kyoto Encyclopedia of Genes and Genomes (KEGG) and Gene Ontology (GO) annotation were implemented using an online annotation server, KOBAS 3.0 (http://kobas.cbi.pku.edu.cn). GO and KEGG enrichment analyses were performed using ClusterProfiler (Yu et al., 2012). *P* values were corrected using Benjamini-Hochberg FDR (false discovery rate). GO and KO terms with a corrected *P* value < 0.05 were treated to be significantly enriched.

Gene enrichment analysis permutations were performed to test whether the enriched GO and kegg terms from outlier genes are likely to be observed when choosing the same number of genes randomly across the genome. For each population comparison, 100 permutations were performed by randomly resampling the same number of genes as outlier genes. We then ran ClusterProfiler for each permutation as described above and counted the number of times the significant GO and kegg terms from the outlier genes also showed up in the randomly selected genes.

## Results

### Growth and Survival Rate in High and Low Elevation Common Gardens

At the high elevation site (Linzhi: LZ), the seedlings of all populations suffered heavy mortality. In contrast, survival rates were comparatively high and constant at the low elevation site (Kunming: KM). By the third year, survival rates produced exceedingly contrasting results with 7.6 vs. 78.2% for the Tibet (LZ) and Kunming (KM) sites, respectively (Zhao et al., 2014). In the seventh year (2017), only five individuals which originated from population KM survived at the high-elevation site (LZ). As natural populations can harbor a great deal of standing genetic variation, these individuals may contain significant divergence in allele frequency contributing to adaptation to high elevation environments. Thus, these individuals were sampled as high elevation selected (survival) population to detect genetic variation related to adaptation to high elevation environments in this study (these results concern the 6-7^th^ year of the common garden experiment).

In addition, significant differences of growth between populations were observed between common garden sites, suggesting adaptive divergence among *P. yunnanensis* natural populations. In the low elevation site (KM), the local population (KM, 249.7 ± 24.2 cm) and two exotic populations from farthest Southwest (BS, 262.9 ± 30.4; GS, 251.5 ± 23.0) generally displayed the fastest growth. The most south population (YX) showed the slowest growth (183.2 ± 26.2), and the populations from the Northwest (ZD, 215.3 ± 13.7; LJ, 221.6 ± 15.4) showed intermediate seedling heights. YL, a population close to the local population (KM), showed slower but medium growth performance (190.1 ± 15.5) in comparison to the local population.

### RNA-Seq and SNP Discovery

We have successfully constructed cDNA libraries for all the 54 sampled individuals. The RNA-seq yielded a total of 2,280 million clean reads, ranging from 30 to 60 million reads for each individual with an average of 42 million reads. On average, 81.93% of the reads were uniquely aligned to the *P. taeda* reference genome v1.01 (**Table S1**). After stringent quality-filtering, we retained a total of 103,608 high quality SNPs captured across the 54 individuals, which involve 8,595 annotated genes in the reference genome. Annotation of these SNPs indicated the presence of 24,439 (23.59% of the total SNPs) synonymous, 23,589 (22.77%) nonsynonymous variants, and 3,515 (3.39%) from 5′ UTR, 5,239 (5.06%) 3′ UTR, 8,089 (7.81%) intronic, 279 (0.27%) splice sites, and 38,458 (37.12%) intergenic mutations (**Table 2**).

**Table 2:** The distribution of full SNPs and outlier SNPs across gene regions.

Class	All SNPs (103,608 SNPs)	Outliers		
		KM_(LZ)_ *vs*. KM-YL_(KM)_ (321 SNPs)	KM_(LZ)_ *vs*. ALL_(KM)_ (294 SNPs)	Overlap (87 SNPs)
Synonymous	24,439 (23.59%)	79 (24.61%)	64 (21.77%)	24 (27.59%)
Nonsynonymous	23,589 (22.77%)	87 (27.10%)	89 (30.27%)	29 (33.33%)
Intronic	8,089 (7.81%)	21 (6.54%)	31 (10.54%)	9 (10.34%)
5′UTR	3,515 (3.39%)	3 (0.93%)	9 (3.06%)	1 (1.15%)
3′UTR	5,239 (5.06%)	22 (6.85%)	3 (1.02%)	1 (1.15%)
Intergenic	38,458 (37.12%)	108 (33.64%)	96 (32.65%)	22 (25.29%)
Splice site	279 (0.27%)	1 (0.31%)	2 (0.68%)	1 (1.15%)

### Population Structure

**Figure 2:**
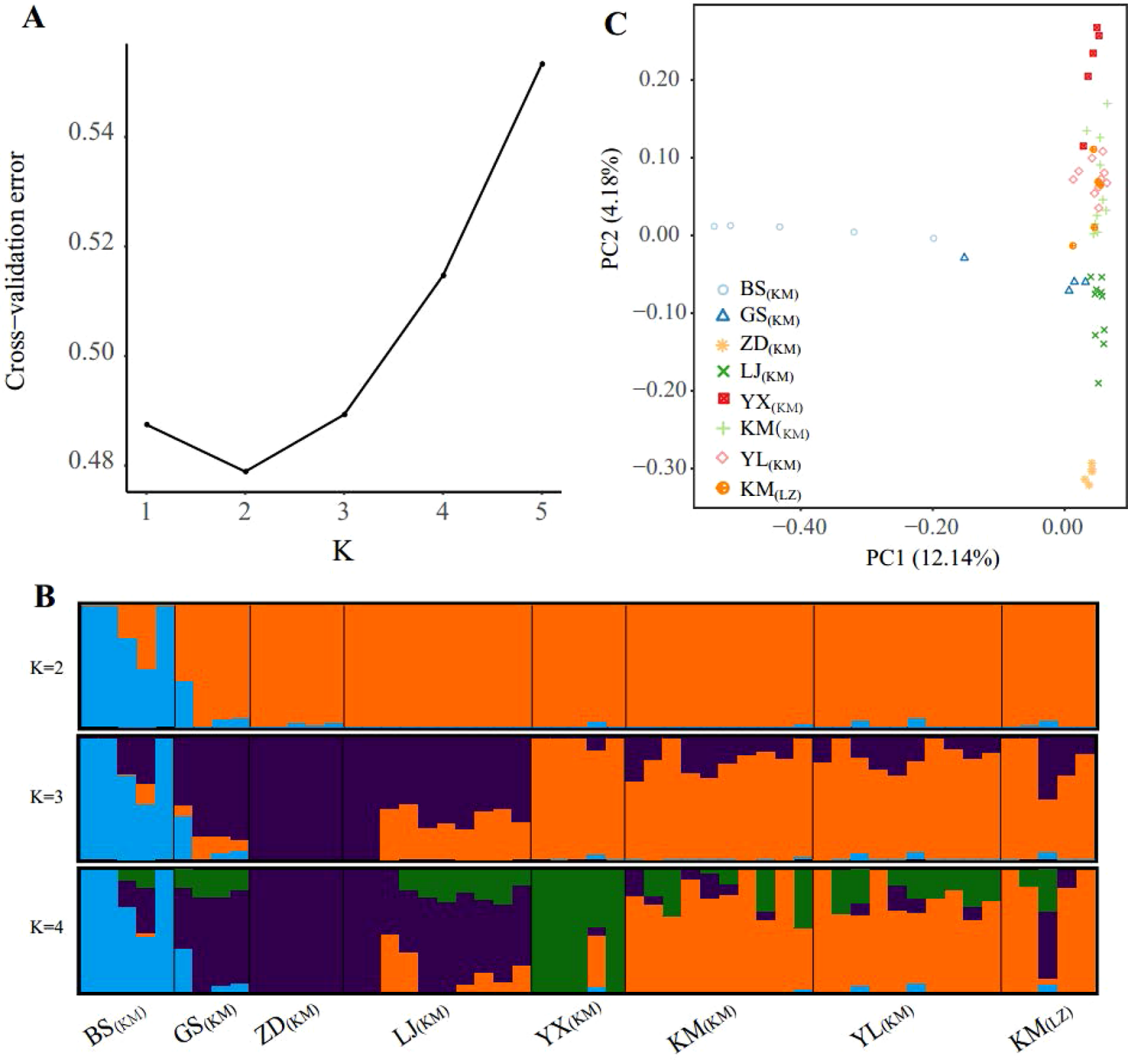
Population structure of the sampled individuals based on full SNP dataset. **(A)** Plot of Cross-validation (CV) error, **(B)** Genetic assignments under K = 2 – 4 based on ADMIXTURE results, **(C)** Plot of the two principal components and the percentage of variance explained resulting from a principal component analysis.

The population structure inferred using the full SNP set (103,608 SNPs) was identical to that from the pruned SNPs (18,329), suggesting little impacts of LD in the ADMIXTURE analysis (**Figures 2B** and **S1**). Cross-validation errors determined that the most likely *K* value is 2 (**Figure 2A**). With *K* = 2, one western marginal population (BS_(KM)_) was split from the other populations. For *K* = 3, three northwestern populations (GS_(KM)_, ZD_(KM)_, and LJ_(KM)_) further formed a northwest cluster and the remaining 3 central populations (KM including KM_(KM)_ and KM_(LZ)_, YL_(KM)_, YX_(KM)_) formed another central cluster. Under the *K* value of 4, population YX_(KM)_ from the most south was further separated from the central cluster. The high-elevation selected populations KM_(LZ)_ and KM_(KM)_ and YL_(KM)_ were in the same genetic cluster under *K =* 2, 3 and 4.

PCA provided further support to the patterns detected by ADMIXTURE (**Figure 2C**). Along the first principal component (PC1: accounting for 12.14% of total genetic variance), BS_(KM)_ was separated from the other populations, supporting the ADMIXTURE finding at *K* = 2. PC2 was accounting for 4.18% of the total variance and showing 3 population clusters, YX_(KM)_, northwest (GS_(KM)_, ZD_(KM)_, and LJ_(KM)_) and central (KM_(KM)_, YL_(KM)_, and KM_(LZ)_), which were distinguished from each other. PCA revealed a continuous genetic differentiation across the *P. yunnanensis* range, consistent with the ADMIXTURE results at *K* = 4.

### Detection of Outlier Loci

**Figure 3:**
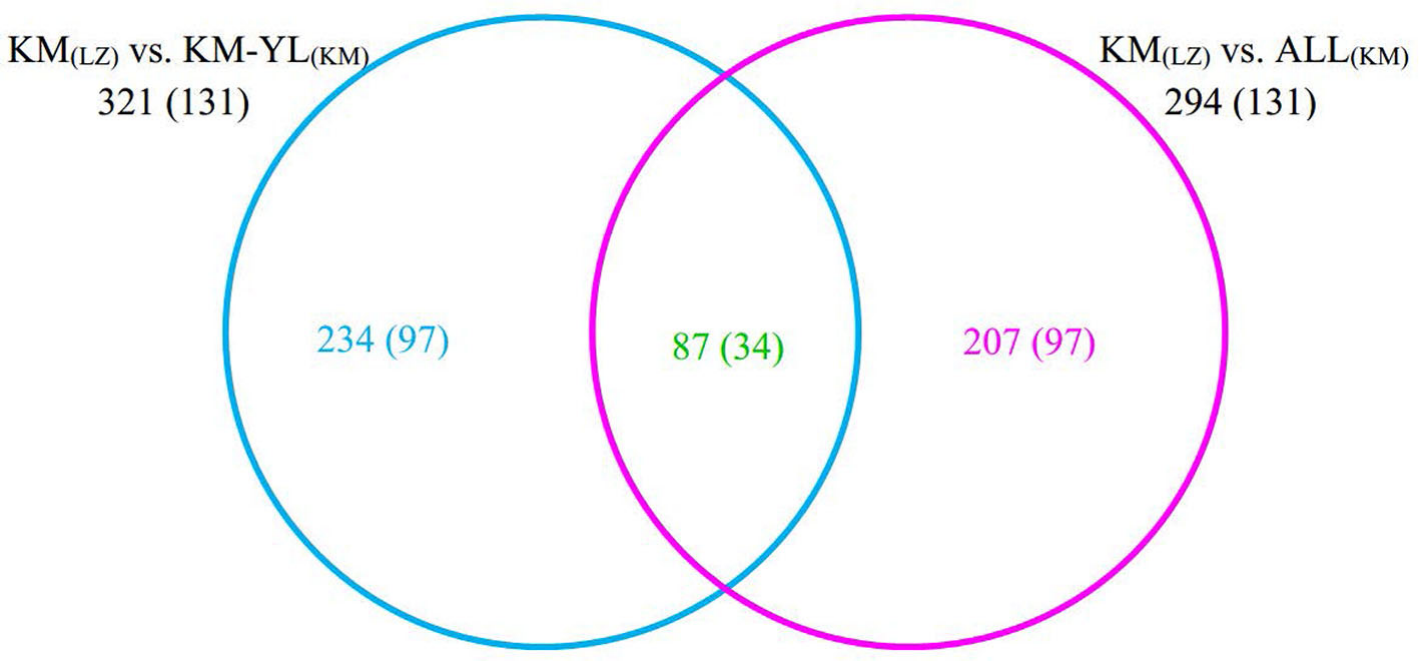
Overlap between outlier SNPs from the two comparisons. The numbers in parentheses indicate the number of genes overlapping with outlier SNPs.

Our *F*_ST_ procedure detected 321 outlier SNPs overlapping 131 genes for the comparison of KM_(LZ)_ *vs.* KM-YL_(KM)_, including 79 (24.61%) synonymous, 87 (27.10%) nonsynonymous, 3 (0.93%) 5′ UTR, 21 (6.54%) intronic, 22 (6.85%) 3′ UTR, 1 (0.31%) splice site, and 108 (33.64%) intergenic mutations (**Tables 2** and **S2**). Additionally, 294 outlier SNPs for the comparison of KM_(LZ)_ *vs.* ALL_(KM)_ were also identified regardless of the impact of population structure which may cause false positives (**Tables S5**–**S7**), with 87 overlaps with the comparison of KM_(LZ)_ *vs.* KM-YL_(KM)_ (**Figure 3**; **Tables S8**–**S10**). The outlier SNP included higher proportion of nonsynonymous mutations (27–33%) compared to the genome average (22%) (**Table 2**). Compared to the whole genomic background, these 131 genes with outlier SNPs between KM_(LZ)_ and KM-YL_(KM)_ showed greater π across coding regions (Wilcoxon rank sum test, *P <*0.05), and slightly higher but not significant d_N_/d_S_ values (Wilcoxon rank sum test; *P* > 0.05) with a mean of 0.4751 and maximum of 2.7400, in the high-elevation selected population KM_(LZ)_ (**Table 3**)_._

**Table 3:** Summary of population diversity statistics. π: nucleotide diversity across coding region for individual gene; d_N_: mean number of pairwise nonsynonymous substitutions per nonsynonymous site; d_S_: mean number of pairwise synonymous substitutions per synonymous site. Outlier 1: outlier genes between KM_(LZ)_ and KM-YL_(KM)._ Outlier 2: outlier genes between KM_(LZ)_ and ALL_(KM)_, Overlap: overlap between outlier 1 and outlier 2.

Population	Site class	π	d_N_	d_S_	d_N_/d_S_
KM_(LZ)_	Whole genome	0.0018	0.0014	0.0047	0.3750
	Outlier 1	0.0026	0.0017	0.0060	0.4751
	Outlier 2	0.0027	0.0021	0.0064	0.4590
	Overlap	0.0026	0.0019	0.0067	0.5962
KM-YL_(KM)_	Whole genome	0.0018	0.0014	0.0047	0.4603
ALL_(KM)_	Whole genome	0.0018	0.0014	0.0048	0.4971

### Functional Annotation

**Figure 4:**
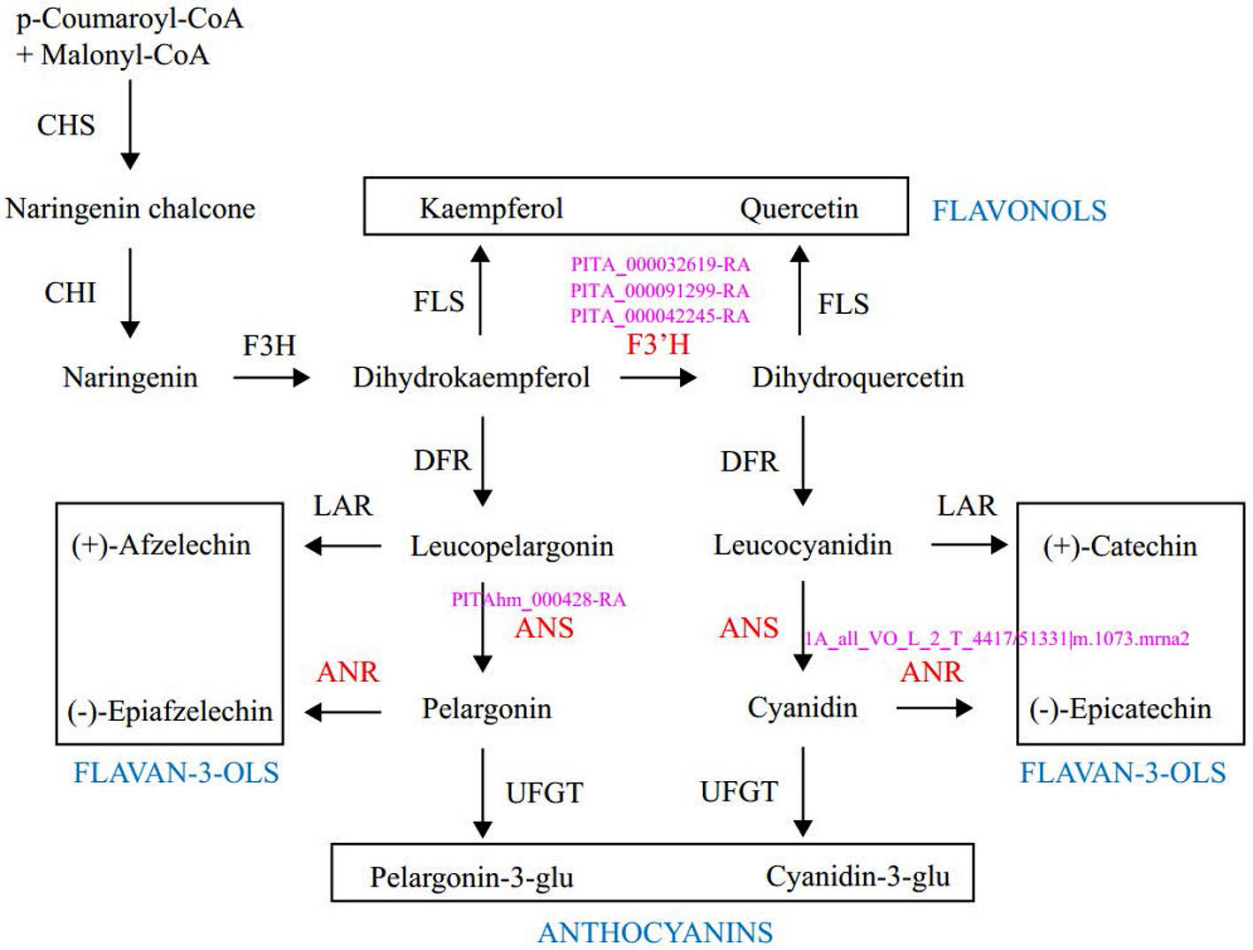
Outlier genes involved in the flavonoid biosynthetic pathway. Enzymes and intermediates are indicated in black. Enzymes in red are identified as targets under divergent selection, with corresponding outlier gene ID in pink. End products are placed in the square. CHS, Chalcone synthase; CHI, chalcone isomerase; F3H, fla-vanone 3-hydroxylase; F3′H, flavonoid-3′-hydroxylase; FLS, flavonol synthase; DFR, dihydroflavonol 4-reductase; LAR, leucoanthocyanidin reductase; ANS, anthocyanidin synthase; ANR, anthocyanidin reductase; UFGT, UDP-glucose: flavonoid 3-O gluco-syltransferase.

Enrichment analysis of 131 outlier genes for the comparison of KM_(LZ)_ *vs.* KM-YL_(KM)_ suggested that five “flavonoid biosynthesis pathway” genes were significantly overrepresented (**Figure 4**; **Table S3**). Of these five genes, three (gene ID: PITA_000032619-RA, PITA_000091299-RA and PITA_000042245-RA) encoded flavonoid 3-hydroxylase (F3'H) that catalyzes the formation of dihydroquercetin from dihydrokaempferol or naringenin. One gene (PITAhm_000428-RA) encoded anthocyanidin synthase (ANS) which catalyzes the oxidation of Leucoanthocyanidins (e.g., leucocyanidin, leucopelargonidin) to colored but unstable anthocyanidins (e.g., cyanidin and pelargonidin); and another one (1A_all_VO_L_2_T_4417/51331|m.1073.mrna2) encoded anthocyanidin reductase (ANR) which converts anthocyanidins to epi-flavan-3-ols (e.g., epicatechin, epigallocatechin) (**Figure 4**). Two SNPs causing nonsynonymous amino changes (**Table 4**) were found in two of the five above-mentioned genes (PITA_000032619-RA and PITA_000042245-RA). Functional analysis of these 131 outlier genes showed that five outlier genes are involved in functional categories associated with high elevation adaptation, including DNA repair (gene ID: PITA_000002229-RA), response to UV (PITA_000032619-RA and PITA_000042245-RA), response to reactive oxygen species (ROS) (PITAhm_000683-RA), and membrane lipid metabolic process (PITA_000060397-RA) (**Tables 4** and **S4**). In addition, 3 outlier genes were annotated as TFs (1 bZIP, 1 NAC and 1 Trihelix). BZIP and NAC are known to be involved in the regulation of secondary metabolism, key components of stress response in plants (Vom Endt et al., 2002) (**Table S2**). Additionally, overlapping outlier genes between the two comparisons were significantly overrepresented in flavonoid biosynthesis pathway (gene IDs: PITA_000032619-RA, PITA_000042245-RA and PITA_000091299-RA), and carbon fixation in photosynthetic pathway (gene IDs: PITA_000042917-RA and PITA_000092661-RA) (**Table S9**), which suggests energy metabolism genes may contribute to adaptation to high elevation environments.

**Table 4:** Outlier SNPs from genes involved in flavonoid biosynthesis pathway and functional categories associated with high elevation adaptation for the comparison of KM_(LZ)_ *vs.* KM-YL_(KM)_.

Scaffold	Position	Reference	Alternate	Effect	Amino change	Gene ID	Arabidopsis gene ID
C32565270	146920	G	A	synonymous		PITA_000032619-RA	AT5G07990
C32565270	146923	C	T	synonymous		PITA_000032619-RA	AT5G07990
C32565270	146932	C	T	synonymous		PITA_000032619-RA	AT5G07990
C32565270	146998	C	T	synonymous		PITA_000032619-RA	AT5G07990
C32565270	147108	C	T	missense	Gly -> Ser	PITA_000032619-RA	AT5G07990
C32565270	147466	G	A	synonymous		PITA_000032619-RA	AT5G07990
scaffold439451	6733	G	A	Upstream_2k		PITA_000091299-RA	AT5G07990
scaffold439451	6754	G	C	Upstream_2k		PITA_000091299-RA	AT5G07990
tscaffold8551	75504	C	T	missense	Gly -> Arg	PITA_000042245-RA	AT5G07990
tscaffold2458	148044	C	G	intron		PITAhm_000428-RA	AT4G22880
tscaffold2325	32570	T	C	Upstream_2k		1A_all_VO_L_2_T_4417/51331|m.1073.mrna2	AT1G61720
scaffold440391	610444	G	A	synonymous		PITA_000002229-RA	AT4G12740
tscaffold1243	243167	G	C	intron		PITAhm_000683-RA	AT1G77120
tscaffold1243	243242	C	T	intron		PITAhm_000683-RA	AT1G77120
C32508606	49016	C	G	synonymous		PITA_000060397-RA	AT3G06460
C32508606	49484	C	G	3_prime_UTR		PITA_000060397-RA	AT3G06460

To test whether the significant terms found within the outlier genes could also be found by chance when sampling genes from the genome, we performed enrichment permutations. None of the significant GO and kegg terms from our outlier analysis for the comparison of KM(LZ) vs. KM-YL(KM) reoccured during our permutations. Thus, we find that the outlier genes differ from the genomic background and that the significant terms are unlikely to be observed by chance.

## Discussion

RNA sequencing allowed the identification of a large number of *P. yunnanensis* SNPs, most of which are from expressed genes. Based on all 103,608 high quality SNPs, a clear population structure emerged with two distinct population clusters comprising one southwestern corner population (BS) and all other populations, in which continuous genetic differentiation was found. These results are in agreement with previous findings that were based on combining mitochondrial and chloroplast DNA markers (Wang et al., 2013).

*F*_ST_ outlier analysis, such as *F*_ST_ scan and BayeScan 2.1 (Foll and Gaggiotti, 2008), is based on the assumption that nonselective processes have the same effect on all loci, while selection would only act on certain loci in the genome. Therefore, loci with very high genetic differentiation (*F*_ST_) are considered to be under divergent selection (Vitti et al., 2013). However, *F*_ST_ outlier analysis presents two main limitations; namely, the high number of false positives produced by chance, and the lack of power to detect true positives; both of them are clearly discussed in the literature (De Mita et al., 2013; Lotterhos and Whitlock, 2014; Haasl and Payseur, 2016). Thus, it is expected that some of these putative outlier SNPs may be false positives produced by chance. Despite these caveats, *F*_ST_ outlier methods have been used in a large number of studies and has brought emblematic allele discoveries at genes involved in adaptation to different environment conditions (Feulner et al., 2015; Wang et al., 2015; Elgvin et al., 2017; Fustier et al., 2017). However, BayeScan has been considered more conservative in identifying outlier SNPs than other methods (Narum and Hess, 2011b; Cuervo-Alarcon et al., 2018; Gros-Balthazard et al., 2019), and its power to detect outliers depends largely on sample size and number of sampled populations (Lotterhos and Whitlock, 2015; Ahrens et al., 2018). Considering that, in this study, only two populations are involved in each comparison, so, we solely used *F*_ST_ scan to detect outlier SNPs. At present, there are two common strategies to reduce false positives. One strategy is to construct prediction with different algorithms, followed by assessing the consistency of signals (Narum and Hess, 2011a; De Mita et al., 2013). The other is to assess consistency of signals across biological replicates (Kawecki and Ebert, 2004; Conte et al., 2012; Tiffin and Ross-Ibarra, 2014). Additionally, in the present study, a randomization procedure (Feulner et al., 2015; Attard et al., 2018), that involved repeated drawing of random samples (100 times), was used to reduce false positives. Using top 1% *F*_ST_ outlier as a cutoff, we initially identified 980 putative outlier SNPs in the comparison of KM_(LZ)_ *vs*. KM-YL_(KM)_. After introducing additional FDR 0.01 by random sampling, we retained 321 outlier SNPs, suggesting that the FDR randomization procedure is potentially effective in reducing the rate of false positives.

Genetic differentiation between populations with different geographic origins have been found in ADMIXTURE and PCA analyses, and the observed differentiation between populations is an important confounding factor for detecting outliers in the comparison of KM_(LZ)_ *vs*. ALL_(KM)_. Considering that SNPs differentiated between KM_(LZ)_ and ALL_(KM)_ but not between KM_(LZ)_ and KM-YL_(KM)_ suggests they may be involved in population differentiation rather than adaptation to high elevation. Thus, we focused only on the results of the comparison of KM_(LZ)_ *vs.* KM-YL_(KM)_. An limitation of our study was the restricted number of samples surviving in the highland. However, we consider this exceedingly high mortality as evidence for maladaptation and thus we treat the results as exploratory in nature and are indicative of genetic variation to high elevation adaptation. Further functional evaluation may provide clearer insights into the genetic responses of *P. yunnanensis* to high elevation environments.

Natural population can harbor a great deal of standing genetic variation. Selection may result in changes of allele frequency of SNPs under selective pressures to rapidly maximize fitness in harsh environment (Hoffmann and Sgrò, 2011). In the present study, we found 321 outlier SNPs with significant divergence in allele frequency between KM_(LZ)_ and KM-YL_(KM)_. Genes with outlier SNPs showed significantly greater π than the genome average across coding regions and slightly higher d_N_/d_S_ values in the high-elevation selected population KM_(LZ)_. These outlier SNPs consist of 79 (24.61%) synonymous, 87 (27.10%) nonsynonymous, 3 (0.93%) 5′ UTR, 21 (6.54%) intronic, 22 (6.85%) 3′ UTR, 1 (0.31%) splice site, and 108 (33.64%) intergenic mutations. Previous studies have shown nonsynonymous, synonymous, and noncoding SNPs can show signatures of selection. Of these outlier SNPs, nonsynonymous substitutions causing amino acid substitutions and protein sequence changes have been usually considered to be the main target of natural selection. However, synonymous SNPs may affect mRNA alternative splicing, mRNA stability, translation kinetics, and protein expression and function, as previously documented (Chamary et al., 2006; Komar, 2007; Kirchner et al., 2017; Bertalovitz et al., 2018). Likewise, SNPs in noncoding regions may also be involved in the regulation of gene expression (Barrett et al., 2012). Thus, noncoding and synonymous SNPs can display a selection signal, either because they are linked to a selection site or are directly selected by natural selection.

The extremely intense UV radiation on the highland may influence plant growth and developmental processes or cause DNA and protein damage (Frohnmeyer and Staiger, 2003). Low temperatures, a major feature of high elevation, can cause lipid peroxidation and reduce fluidity of lipid membranes by causing fatty acid unsaturation, altering lipid composition and ratio of lipids to proteins in cell membrane (Wang et al., 2006). Both cold stress and strong UV radiation result in the oxidative stress due to generation of reactive oxygen species (ROS), such as hydrogen peroxide, superoxide anion, and hydroxyl radical (Halliwell, 2007; Wang et al., 2010; Lidon and Ramalho, 2011). Previous studies revealed that the DNA repair and radiation responses pathways may contribute to highland adaptation of the *Crucihimalaya himalaica* (Qiao et al., 2016; Zhang et al., 2019), Tibetan highland barley (Zeng et al., 2015), Tibetan antelope (Ge et al., 2013), Tibetan chicken (Zhang et al., 2016), and Tibetan hot-spring snake (Li et al., 2018). In the present study, we found that many outlier genes are involved in response to UV, DNA repair, response to ROS, and membrane lipid metabolic process. This may suggest *P. yunnanensis* adaptation to strong UV radiation and low temperature environments on the highland.

The strong UV-absorbing characteristics of flavonoids have been considered as a primary role to protect plant tissues from high energetic UV. Moreover, many studies have provided new evidence that UV light induces the synthesis of flavonoids (Ryan et al., 2002; Berli et al., 2010; Stracke et al., 2010; Agati et al., 2011; Kusano et al., 2011). These flavonoids can perform antioxidant roles by suppressing and scavenging free radicals such as ROS (Agati et al., 2012), as well as chelating metal ions such as iron, copper, zinc, and aluminum that generate ROS *via* the Fenton reaction (Williams et al., 2004). In addition, they are able to prevent the peroxidation of lipids and the oxidative damage of membrane lipids (Kumar and Pandey, 2012). The central pathways for flavonoid biosynthesis are highly conserved and well characterized (Holton and Cornish, 1995; Winkel-Shirley, 2001; Petrussa et al., 2013; Li et al., 2015). In the present study, outlier genes from the comparison of KM_(LZ)_ *vs.* KM-YL_(KM)_ were significantly enriched for flavonoid biosynthesis pathway with 5 outlier genes coding 3 key enzymes. And this pathway has been shown to help withstand harsh environments in high elevations (Zhang et al., 2003; Casati and Walbot, 2010; Agati et al., 2012). These results suggest that flavonoid biosynthesis pathway may play a key role in the adaptation of *P. yunnanensis* to high elevation environments. Additional functional and physiological experiments are needed to verify the contributions of these genes to high elevation adaptation (Pavlidis et al., 2012).

## Conclusions

We used RNA-seq and *F*_ST_ scan to identify genetic variation related to high elevation adaptation in *P. yunnanensis* by contrasting a high-elevation selected population and low-elevation population sampled from highland and lowland common gardens. Our study provided a genome-wide evaluation of nucleotide diversity in this species, and identified variants and genes that could be involved in adaptation to high elevation environment. Our results suggest that the flavonoid biosynthesis pathway is likely an important selection target, which may play a key role in the adaptation to high elevation environments in *P. yunnanensis*. While these results are based on a small sample size, thus we consider this work as exploratory in nature and further research on high elevation adaptation is warranted.

## Data Availability Statement

All RNA-seq data sets used in this study have been submitted to the NCBI SRA database under Bioproject Accession no. PRJNA545862.

## Author Contributions

JM designed the study. YS, WZ, CX and YX completed the settlement of common garden experiment and data and sample collection. YS and WZ analyzed data. YS wrote the draft. JM, YE-K, and AT revised the manuscript. All authors reviewed the final version of the manuscript.

## Funding

This research was funded by the National Natural Science Foundation of China (NO. 31670664 and 31800550) and Fundamental Research Funds for the Central Universities (2018BLCB08).

## Conflict of Interest

The authors declare that the research was conducted in the absence of any commercial or financial relationships that could be construed as a potential conflict of interest.

## Supplementary Material

Figure S1 Population structure of the sampled individuals based on the thinned SNP dataset. **(A)** Plot of Cross-validation (CV) error. **(B)** Genetic assignment under K = 2 – 4.
