# Supplementary figures and images for: Genetic Variation Related to High Elevation Adaptation Revealed by Common Garden Experiments in Pinus yunnanensis
Source: Front Genet. 2020 Feb 11;10:1405. doi: 10.3389/fgene.2019.01405 (PMC7027398; doi:10.3389/fgene.2019.01405)

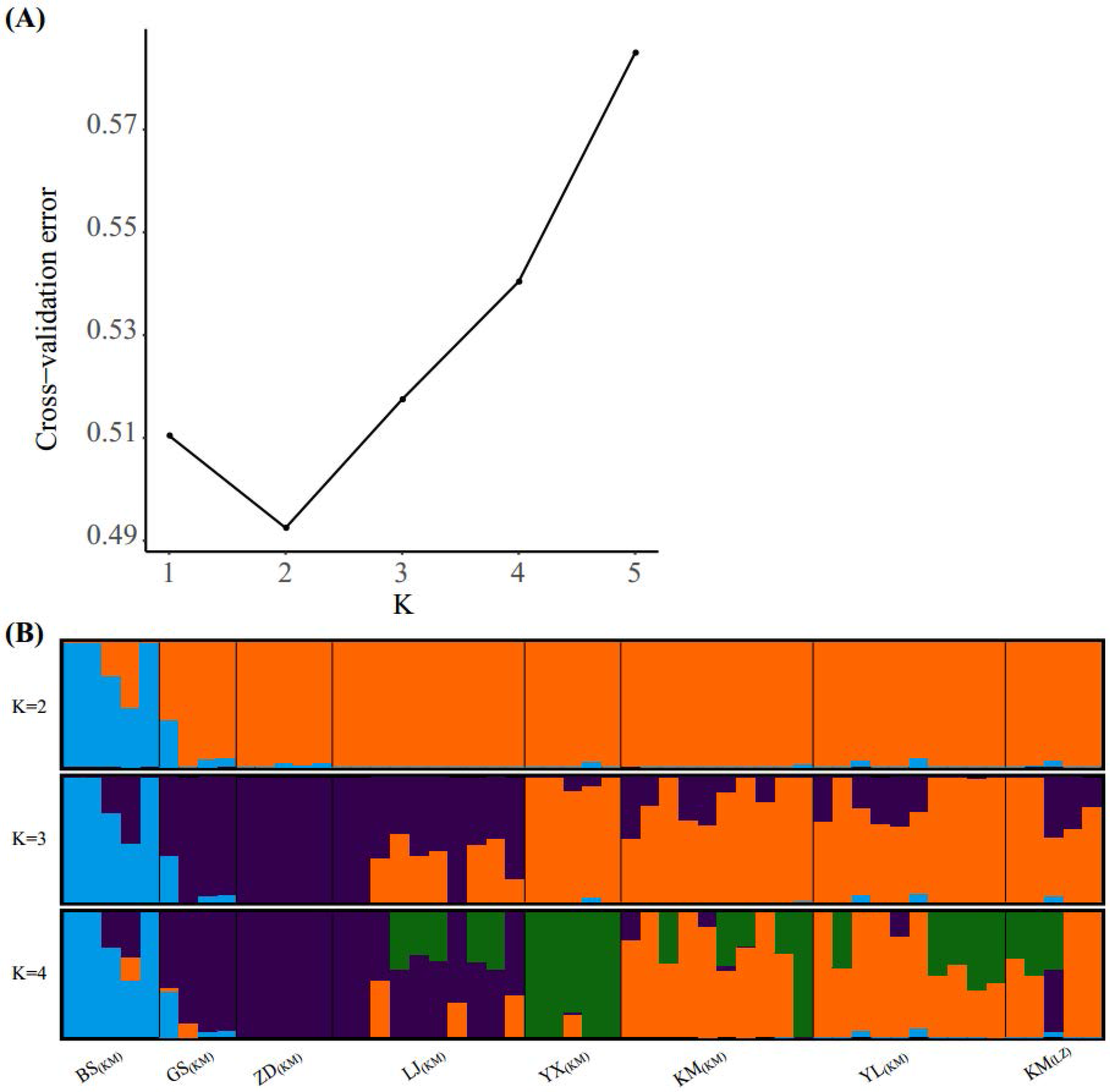

Supplement: Figure S1 — Population structure of the sampled individuals based on the thinned SNP dataset. (A) Plot of Cross-validation (CV) error. (B) Genetic assignment under K = 2 – 4. [file Image_1.tif]
